# Conservation and evolution of the programmed ribosomal frameshift in *prfB* across the bacterial domain

**DOI:** 10.1128/mbio.01055-25

**Published:** 2025-08-18

**Authors:** Cassidy R. Prince, Isabella N. Lin, Heather A. Feaga

**Affiliations:** 1 Department of Microbiology, Cornell University 5922, Ithaca, New York, USA; Indiana University Bloomington 1771, Bloomington, Indiana, USA

**Keywords:** translation termination, RF2, *prfB*, programmed ribosomal frameshifting, ribosome, evolution, Actinomycetota

## Abstract

When the ribosome reaches a stop codon, translation is terminated by a release factor. Bacteria encode two release factors, RF1 and RF2. In many bacteria, the gene encoding RF2 (*prfB*) contains an in-frame premature stop codon near the beginning of the open reading frame. A programmed ribosomal frameshift is therefore required to translate full-length RF2. While the molecular mechanism of the programmed ribosomal frameshift has been extensively characterized in *Escherichia coli*, bioinformatic analysis of the evolution and conservation of this motif has been limited to few genomes. By analyzing >12,000 bacterial genomes, we sought to thoroughly characterize the conserved frameshifting elements within the programmed frameshift motif and identify genomic features of phyla that have lost the motif altogether. We find that the programmed ribosomal frameshift in *prfB* was likely present in the last common ancestor of bacteria and that the motif elements are almost completely conserved, including the identity of the premature stop codon. We find that loss of the programmed frameshift motif is highly correlated with RF2-specific stop codon usage, suggesting that stop codon usage has shaped the conservation of this regulatory mechanism. In support of this model, the programmed frameshift in *prfB* is entirely absent in Actinomycetota, which have particularly high RF2-specific stop codon usage. Finally, we show that a model member of Actinomycetota fails to produce full-length RF2 when provided with an allele of *prfB* that contains the programmed frameshift motif. Altogether, our work provides a thorough characterization of RF2 regulation across the bacterial domain.

**IMPORTANCE:**

Translation termination is catalyzed by one of two release factors in bacteria, RF1 or RF2. It has been known for decades that RF2 levels in *Escherichia coli* are regulated by a programmed ribosomal frameshift within the *prfB* gene that encodes RF2. We investigated the conservation and features of the *prfB* programmed ribosomal frameshift in >12,000 genomes across the bacterial domain. Our data suggest this autoregulatory motif was present in the common ancestor of bacteria and that organisms that lost the motif have high RF2-specific stop codon usage. We also find that overexpression of RF2 from *prfB* lacking the programmed frameshift motif is toxic to *Bacillus subtilis,* as has been observed in *E. coli*. The fitness cost of RF2 overexpression in distantly related species coupled with its broad conservation suggests that RF2 autoregulation imparts a strong selective advantage in organisms that do not have high RF2-specific stop codon usage.

## INTRODUCTION

Ribosomes translate mRNA into protein by iteratively decoding one codon at a time. Reading frame maintenance is essential for translation fidelity. Ribosomal frameshifting alters the identity of downstream amino acids and typically leads to premature termination since the ribosome quickly encounters a stop codon when it deviates from an open reading frame (1). In rare cases, ribosomal frameshifting is required to make a full-length protein (2–5). These types of frameshifts are referred to as programmed ribosomal frameshifts (6–9) reviewed in reference 10.

**Fig 1:**
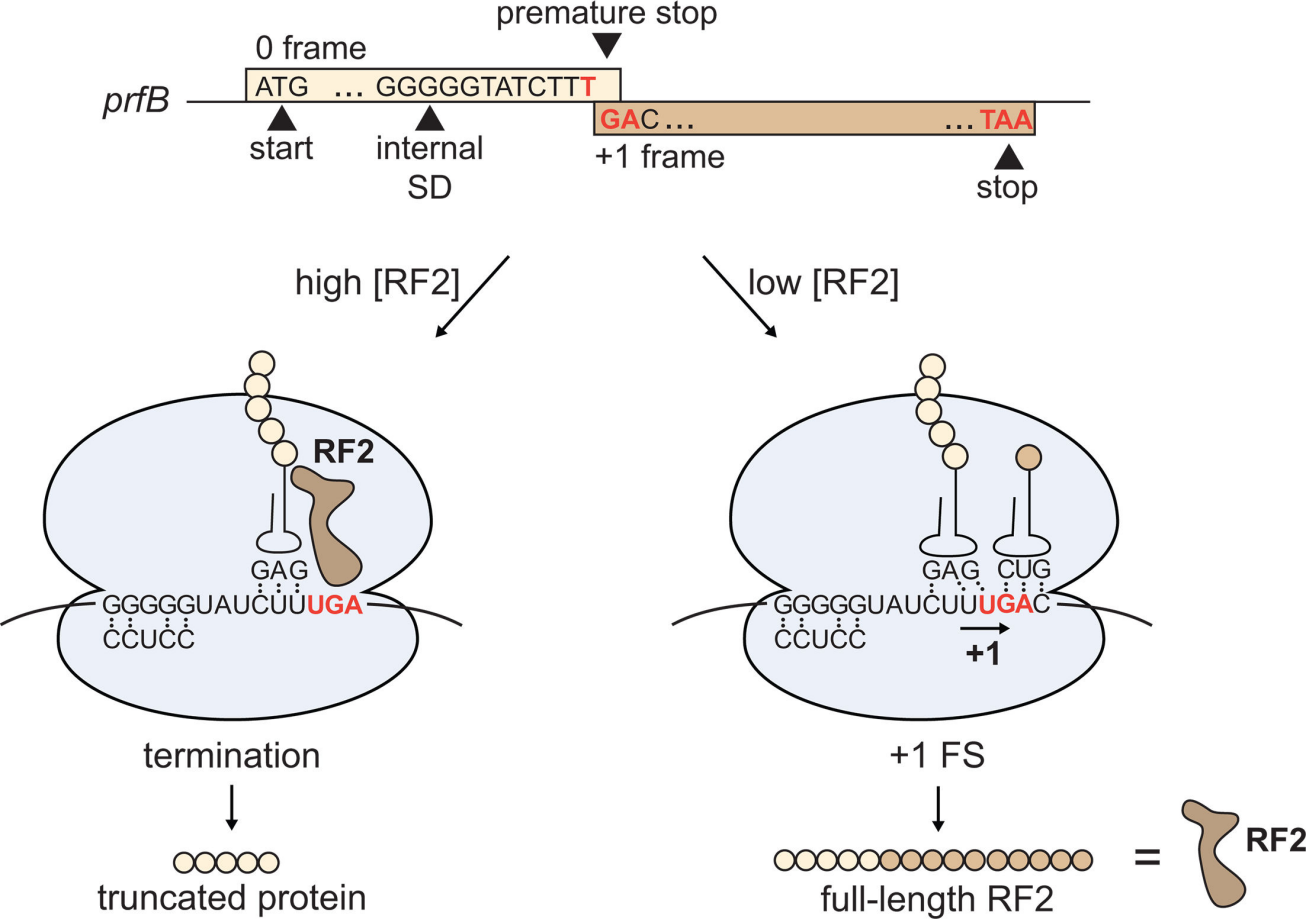
Schematic of *prfB* programmed ribosomal frameshift mechanism. (Top) DNA sequence elements of the *prfB* gene sequence in *E. coli*. (Bottom) The ribosome pauses at the internal SD-like sequence GGGGG, which base-pairs with the anti-SD sequence on the small ribosomal subunit. If RF2 levels are high, RF2 will enter the A-site and terminate translation at the premature TGA stop codon. If RF2 levels are low, the ribosome will slip into the +1 reading frame at the slippery sequence CTTT, causing a GAC codon to be positioned in the A-site instead of the premature stop codon. Therefore, ribosomal frameshifting results in production of full-length RF2.

A textbook example of programmed ribosomal frameshifting in bacteria is found in *prfB*, the gene that encodes release factor 2 (RF2) (11, 12). RF2 terminates translation at TGA and TAA stop codons (13). In *Escherichia coli prfB*, an in-frame TGA codon at position 26 interrupts the open reading frame (11) (Fig. 1). A purine-rich, internal Shine-Dalgarno (SD)-like sequence is located six nucleotides upstream of this stop codon, and a CTT leucine codon immediately precedes the stop codon (14). Together, the CTT leucine codon and the first nucleotide of the TGA stop codon create a contiguous run of four pyrimidines (CTTT), which is referred to as the “slippery sequence.” The TGA stop codon is followed by a C nucleotide, making it a weak stop codon (15–17). Alteration of any of these elements reduces frameshifting at the premature stop codon (14). *In vitro* and *in vivo* studies using *E. coli* show that when concentrations of RF2 are high, RF2 terminates translation at the premature stop codon, creating a truncated peptide (12, 18). Alternatively, when RF2 concentrations are low, the ribosome pauses at the stop codon, and the SD-like sequence in the mRNA base-pairs with the anti-SD at the 3′ end of the 16S rRNA, displacing the E-site tRNA (14, 18, 19). The leucine tRNA in the P site then slips into the +1 frame at the slippery sequence CTTT, causing the ribosome to bypass the premature stop codon and continue translation to produce full-length RF2 (12, 14). Since RF2 levels directly regulate the translation of the *prfB* transcript via termination at the RF2-specific premature stop codon, this frameshifting mechanism autoregulates RF2 levels.

The first bioinformatic analysis of the conservation of the frameshift motif was performed by Persson and Atkins in 1998, with 20 bacterial *prfB* sequences (20), followed by Baranov et al. in 2002 (21). This foundational work determined that approximately 70% of these *prfB* sequences required the programmed ribosomal frameshift to produce RF2, and that in all but one sequence, the in-frame stop codon interrupting the *prfB* open reading frame was TGA. The slippery sequence (CTTT) and the C nucleotide immediately following the stop codon were all highly conserved (21). Follow-up on a larger set of 259 genomes uncovered the regulatory frameshift in 87% of these genomes, indicating that the prevalence of this frameshift was even more widespread than previously determined (22).

Hundreds of thousands of bacterial genomes are now available, facilitating more thorough analyses of the conservation of the *prfB* programmed frameshift as an autoregulatory mechanism. Our analyses are also aided by recent developments in bacterial phylogeny, which better resolve the bacterial species tree. Using a diverse and comprehensive genome set, we sought to answer remaining questions about the evolution and conservation of the programmed frameshift autoregulatory mechanism in *prfB*. For example, was the programmed frameshift present in the ancestral *prfB*, and what are the genome characteristics that are associated with absence of the programmed frameshift?

We surveyed 12,753 bacterial genomes across 21 phyla to determine the prevalence and conserved features of programmed ribosomal frameshifting in *prfB*. We find that most bacterial genomes (64%) encode a *prfB* that requires a ribosomal frameshift to produce full-length RF2 and that the programmed frameshift was most likely present in the common ancestor of bacteria. In our data set, the frameshift sequence motif is extremely well-conserved, and most strikingly, the identity of the premature stop codon is nearly always an RF2-specific TGA stop codon. We did not find any instance of the RF1-specific TAG stop codon within the motif, indicating that the autoregulatory role of the programmed ribosomal frameshift in *prfB* is conserved in taxa that employ this mechanism. In further support of the necessity of RF2 autoregulation, we found that RF2 overexpression is toxic to *Bacillus subtilis*. Next, we examined the species that lack the autoregulatory frameshift motif and found that they have significantly higher RF2 stop codon usage, which may explain why RF2 is not autoregulated by this mechanism in these organisms. Consistent with this model, the actinomycete *Mycobacterium smegmatis*, which has high RF2 stop codon usage, exhibits low frameshifting efficiency at the motif. Cumulatively, our results support the autoregulatory function of the *prfB* frameshift across the bacterial domain and suggest that this autoregulatory mechanism was present in ancestral *prfB*, and that this autoregulation was lost in phyla that exhibit high RF2-specific stop codon usage.

## RESULTS

### Sequence elements of the programmed frameshift within *prfB* are hyper-conserved

To determine the prevalence of the *prfB* programmed frameshift motif, we analyzed the *prfB* sequences of 12,753 representative bacterial species genomes from the NCBI RefSeq database as annotated by NCBI (23). We identified the *prfB* sequence in each of these genomes and determined whether they contained a premature, in-frame stop codon within the *prfB* reading frame. Of the 12,753 genomes surveyed, 8,164 (64%) contain a premature stop codon in *prfB*, indicating that these organisms require a programmed ribosomal frameshift to produce functional RF2.

**Fig 2:**
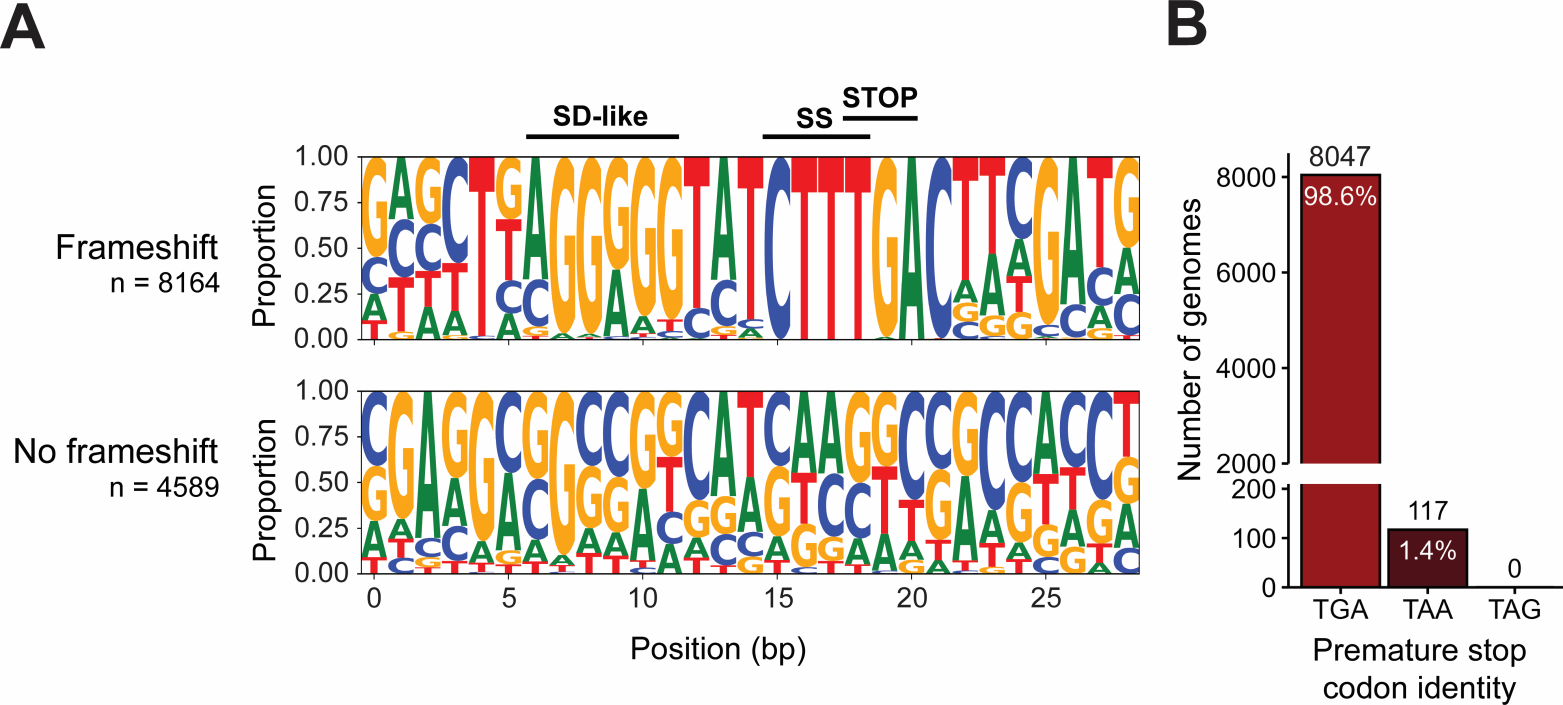
The premature stop codon in the *prfB* frameshift motif is recognizable by RF2 in 100% of genomes surveyed. (**A**) Sequence logos of *prfB* frameshift motif regions for genomes with and without a premature stop codon in *prfB*. The “No frameshift” logo captures the region in which the frameshift motif is expected based on a full alignment of all *prfB* sequences. Labels above the black lines correspond to the following frameshift motif components: SD-like, Shine-Dalgarno-like sequence; SS, slippery sequence; STOP, premature stop codon. (**B**) Identity of the premature stop codon within the *prfB* frameshift motif.

We aligned the *prfB* sequences to generate a nucleotide sequence logo of the programmed frameshift motif (Fig. 2A). Within this motif, the purine-rich internal Shine-Dalgarno-like sequence is hyper-conserved, with the consensus sequence approaching AGGGGG (Fig. 2A). An even more highly conserved slippery sequence of CTTT occurs three nucleotides downstream of the internal SD-like sequence. The cytosine following the premature stop codon is also highly conserved, likely because TGAC causes poor termination efficiency and would permit more frequent frameshifting (15). This canonical CTTTGA sequence is present in nearly every *prfB* frameshift motif. Exceptions included 21 genomes with a long poly-thymine tract in the slippery sequence, in which tRNA^Phe^ would decode the codon before the stop codon instead of tRNA^Leu^ (i.e., TTTTTGA). These genomes belong to predominantly low-GC organisms (mean GC of 35%) in Aquificota and Gammaproteobacteria. In these genomes, the *prfB* sequence retains the internal SD-like sequence as well as the spacing between the SD-like sequence and the slippery sequence, and the identity of the premature stop codon is TGA.

We also aligned the *prfB* sequences of species that do not contain a premature stop codon to determine whether these sequences retained any elements of the programmed frameshift motif. In these *prfB* sequences, the internal SD-like sequence and slippery sequence are not found in the analogous region of the sequence, suggesting that these elements are lost in organisms that encode a fully in-frame RF2 (Fig. 2A). This observation is expected since these elements would still promote ribosomal frameshifting, but without the presence of the autoregulatory TGA stop codon.

Autoregulatory programmed ribosomal frameshifting has been shown in *prfB*, but not in *prfA,* the gene that encodes release factor 1 (RF1) (11, 22). To serve as a comparator, we performed the same analysis on *prfA* with genomes from the same data set. Only 34 genomes have a copy of *prfA* that is annotated as a “pseudogene” and would require a frameshift for RF1 production with no alternative functional *prfA* (Table S1). The location of the premature stop codon is inconsistent between species; there are no apparent conserved frameshifting elements around the premature stop codons, and the species with *prfA* pseudogenes are phylogenetically distant from each other. Therefore, it is unlikely that programmed frameshifting is used to regulate RF1 in these genomes. Additionally, most of the genomes with *prfA* pseudogenes are the only genome of their species. More genome sequences from these species are necessary to determine whether *prfA* is genuinely a pseudogene in these species or whether these cases are sequencing errors.

### The RF1-specific TAG stop codon is not detected as the premature stop codon in the *prfB* programmed frameshift motif

Bacteria terminate translation using one of three stop codons: TAA, TAG, or TGA. TAA is recognized by either RF1 or RF2, whereas TAG is RF1-specific and TGA is RF2-specific (13). To assess the conservation of the RF2-specific stop codon within the programmed frameshift motif, we determined the identity of the premature in-frame stop codon in the 8,164 genomes that contain the motif. We found that 98.6% of genomes with the motif contain the RF2-specific TGA stop codon as the premature stop codon (CTTTGA) (Fig. 2B). A total of 1.4% of genomes contain a TAA premature stop codon (CTTTAA). Since RF2 terminates translation at TAA as well as TGA, RF2 expression in these taxa would also be sensitive to the levels of RF2 in the cell. Genomes encoding TAA as the premature stop codon are observed in multiple phyla and retained among strains of a species (Fig. S1). These findings suggest that the TAA codon is poorly tolerated since it has not become broadly fixed throughout particular clades and that the premature TGA is preferred for RF2 autoregulation (Fig. 2B). Most strikingly, none of the 8,164 genomes encode an in-frame RF1-specific TAG stop codon. The ubiquity of TGA as the premature stop codon in the motif and the absence of TAG suggest that the universal purpose of the programmed frameshift motif is indeed RF2 autoregulation.

### The programmed frameshift in *prfB* was likely present in the last common ancestor of bacteria

**Fig 3:**
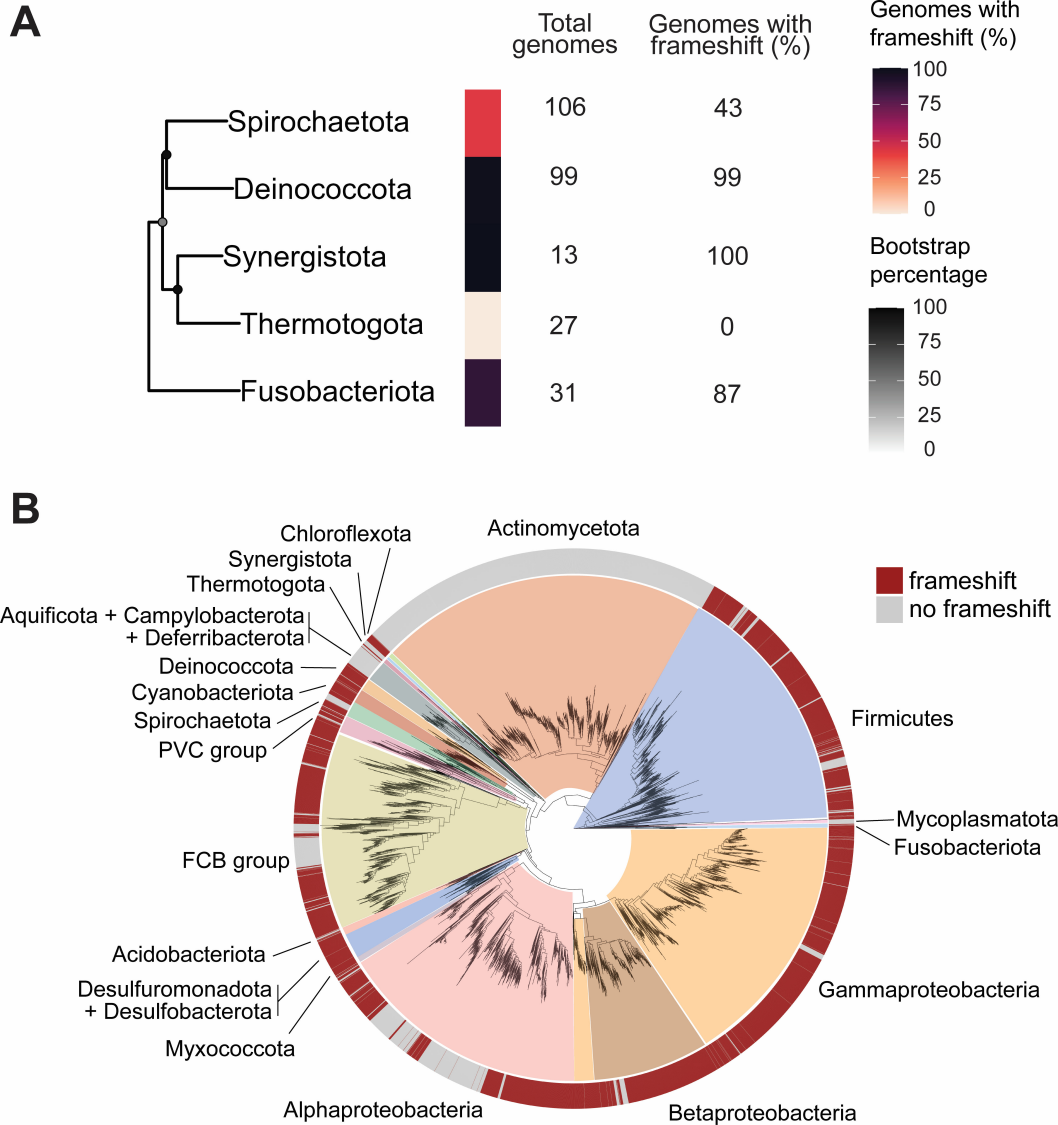
The *prfB* frameshift motif is broadly distributed across bacterial phyla. (**A**) Percent of genomes containing the *prfB* frameshift motif within phyla most closely related to the last bacterial common ancestor. A subtree of the large 16S tree was created using a random single representative genome for each phylum. Values for the number of analyzed genomes and percent of genomes with the frameshift per phylum are located to the right of each bar. Darkened circles indicate bootstrap values for each node. (**B**) A 16S maximum-likelihood phylogenetic tree showing distribution of genomes that encode the programmed frameshift within *prfB*. Phyla with more than 10 available and high-quality reference genomes are labeled.

Bioinformatic analyses of the programmed frameshift motif in *prfB* sequences by Baranov et al. found that the motif is absent in *Aquifex aeolicus* and *Thermotoga maritime* (24). At the time, these taxa were thought to be the deepest branching taxa and most closely related to the ancestor of eubacteria. Therefore, the absence of the programmed frameshift in the *prfB* of *A. aeolicus* and *T. maritime* suggested it may have been absent in the ancestral *prfB*. Recent efforts have rooted the bacterial tree in the neighborhood of Fusobacteriota (25). This neighborhood also includes the Spirochaetota and a clade comprising Deinococcota, Synergistota, and Thermotogota (DST clade). Consistent with Baranov et al., we did not detect the programmed frameshift in any of the 27 Thermotogota genomes now available (Fig. 3A). However, we detected the programmed frameshift in >85% of *prfB* sequences from most other lineages in this neighborhood. The programmed frameshift is present in 87% of *prfB* sequences from Fusobacteriota (*n* = 31), 100% of Synergistota (*n* = 13), 99% of Deinococcota (*n* = 99), and 42% of Spirochaetota (*n* = 106) (Fig. 3A). The presence of the programmed frameshift motif in taxa closest to the root of the bacterial tree suggests that the ancestral *prfB* contained the programmed frameshift.

Next, we determined the distribution of the *prfB* programmed frameshift motif throughout the bacterial domain (Fig. 3B; Fig. S2). Only three phyla with >10 available genomes completely lacked the frameshift motif: Actinomycetota, Mycoplasmatota, and Thermotogota. In 14 out of 19 phyla with >10 available genomes, >50% of genomes contain the premature stop and frameshift motif (Fig. S2), demonstrating strong conservation of the programmed frameshift. For phyla where most genomes contain the motif, it is likely and parsimonious that the common ancestor of the phylum contained the motif, and that the motif was lost in recent lineages. Altogether, our results suggest that the programmed ribosomal frameshift in *prfB* is widespread throughout bacteria, including in deeply rooted branches of the bacterial domain.

### Both free-living and obligate intracellular species retain the *prfB* programmed frameshift motif

Alphaproteobacteria are unique in that about 50% of genomes in this phylum encode the programmed ribosomal frameshift motif within *prfB* (Fig. S2). Since this phylum comprises both free-living and obligate intracellular bacteria, we questioned whether intracellular species would be less likely to autoregulate RF2 levels with the programmed ribosomal frameshift. We observed that Alphaproteobacterial genomes that do not use the frameshift motif in *prfB* fall predominantly in two orders: Rhodobacterales (*n* = 486 genomes) and Sphingomonadales (*n* = 338 genomes). Within Rhodobacterales, only two genomes appear to utilize the programmed frameshift motif. These were the only genomes of their respective genera, so additional genomes for the species could not be consulted to rule out sequence inaccuracy. Within Sphingomonadales, only 15 genomes contained the frameshift motif, and these genomes were in the family Sphingosinicellaceae. Members of both Rhodobacterales and Sphingomonadales tend to be free-living and are found in soils, water, and sediments (26). Other free-living orders of the Alphaproteobacteria, such as Caulobacterales and Rhodospirillales, predominantly encode the programmed frameshift motif in *prfB*. Meanwhile, the obligate intracellular Alphaproteobacterial genera including *Rickettsia* and *Wolbachia* maintain the *prfB* gene and its frameshift motif. Therefore, we conclude that the programmed frameshift motif is conserved in both free-living and obligate intracellular species within the Alphaproteobacteria.

### Overexpression of RF2 is toxic to *Bacillus subtilis*

**Fig 4:**
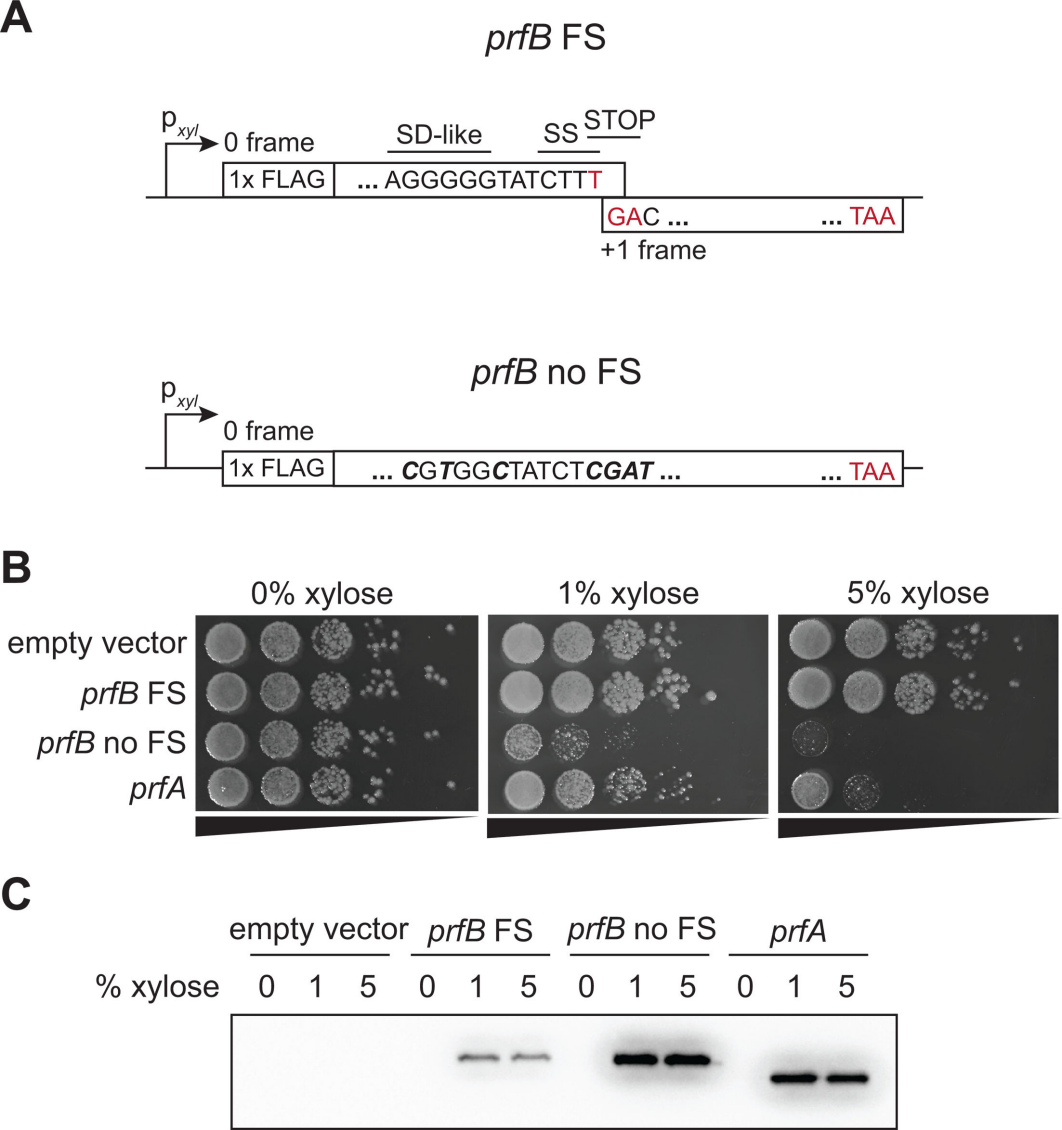
Overexpression of RF2 without the autoregulatory programmed frameshift motif is toxic to *B. subtilis*. (**A**) Schematic of *prfB* overexpression vectors in *B. subtilis*. The “*prfB* FS” construct maintains all elements of the autoregulatory *prfB* frameshift motif. In the “*prfB* no FS” construct, the internal SD-like sequence, slippery sequence, and in-frame stop codon have been mutated to eliminate all key elements of the motif and ensure production of full-length RF2. The exact base changes that were made are bolded and italicized. (**B**) *B. subtilis* cells overexpressing *prfB* were serially diluted and plated on varying levels of xylose for induction of *prfB* variant overexpression. Spot plates are representative of three independent biological replicates. (**C**) Western blot of FLAG-tagged full-length RF2 variant and RF1 levels during overexpression.

The extreme conservation of the RF2-specific premature stop codon in the *prfB* programmed frameshift motif suggests that autoregulation of RF2 levels imparts a strong selective advantage. In *E. coli*, even a minor threefold increase in RF2 is sufficient to show a modest growth defect (27). *In vitro* and *in vivo E. coli* studies show that increased release factor concentration leads to increased premature termination at sense codons (27, 28). To test whether RF2 overexpression has a deleterious effect in a Gram-positive organism that is evolutionarily distant from *E. coli*, we expressed a variant of *prfB* lacking the premature in-frame stop codon under the control of a xylose-inducible promoter in *Bacillus subtilis* (Fig. 4A). Cells that expressed this construct failed to form colonies on plates at 30°C (Fig. 4B), whereas cells that expressed wild-type *prfB* containing the programmed frameshift motif grew similarly to cells containing empty vector (Fig. 4B). These results indicate that uncontrolled expression of *prfB* has a negative effect on fitness, and that the autoregulation imparted by the frameshift motif is sufficient to control RF2 expression. For comparison, we also overexpressed RF1 from the same promoter. We found that comparable levels of RF1 overexpression also reduce fitness, but to a far lesser extent than RF2 overexpression (Fig. 4B and C).

### The *prfB* programmed frameshift motif is absent in Actinomycetota and Mycoplasmatota

It was previously hypothesized that the lineages of Mycobacteria and Streptomyces lost the programmed frameshift in *prfB* based on its absence in two species*—Mycobacterium tuberculosis* and *Streptomyces coelicolor* (20). Expanding upon this, we surveyed the *prfB* sequences of the Actinomycetota phylum that includes these lineages and did not detect the programmed frameshift in any of these genomes (*n* = 2,658) (Fig. 3B), supporting a loss event in the common Actinomycetota ancestor.

**Fig 5:**
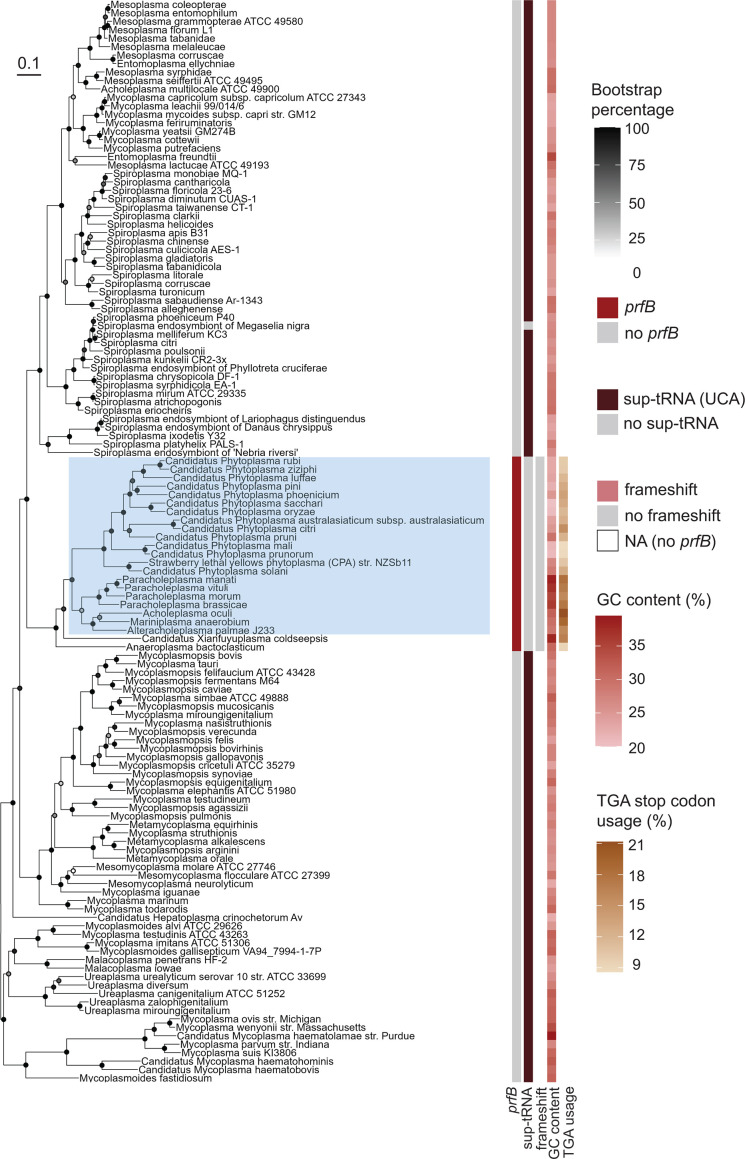
Most Mycoplasmatota species lack *prfB* and suppress TGA stop codons. Core SNP maximum-likelihood tree of 128 Mycoplasmatota species. From left to right, columns represent *prfB* gene presence, TGA stop codon suppressor tRNA presence, *prfB* frameshift motif presence, GC content, and TGA stop codon usage. The Acholeplasmataceae family is highlighted in blue. Darkened circles indicate bootstrap values for each node.

Our analysis also indicates that the *prfB* programmed frameshift was likely absent in the common ancestor of the Mycoplasmatota phylum. Mycoplasmatota species are most closely related to Firmicutes (29), but have significantly reduced genome sizes, low GC content (30), and particularly low TGA stop codon usage (Fig. 5). Many species within Mycoplasmatota lack the *prfB* gene completely (31, 32) and utilize nearly zero TGA stop codons (33). Instead, TGA codons are decoded as tryptophan by suppressor tRNAs (30, 32, 34, 35). We found that all genomes lacking *prfB* encoded a suppressor tRNA for the TGA stop codon (Fig. 5). Only 23 out of 128 Mycoplasmatota genomes retained *prfB,* and none of these sequences contain the programmed frameshift motif (Fig. 5). Species that retain *prfB* are predominantly found within the Acholeplasmataceae family (Fig. 5), including important *Phytoplasma* plant pathogens (36).

### Genomes that lack the programmed frameshift motif in *prfB* have higher GC content and TGA stop codon usage

**Fig 6:**
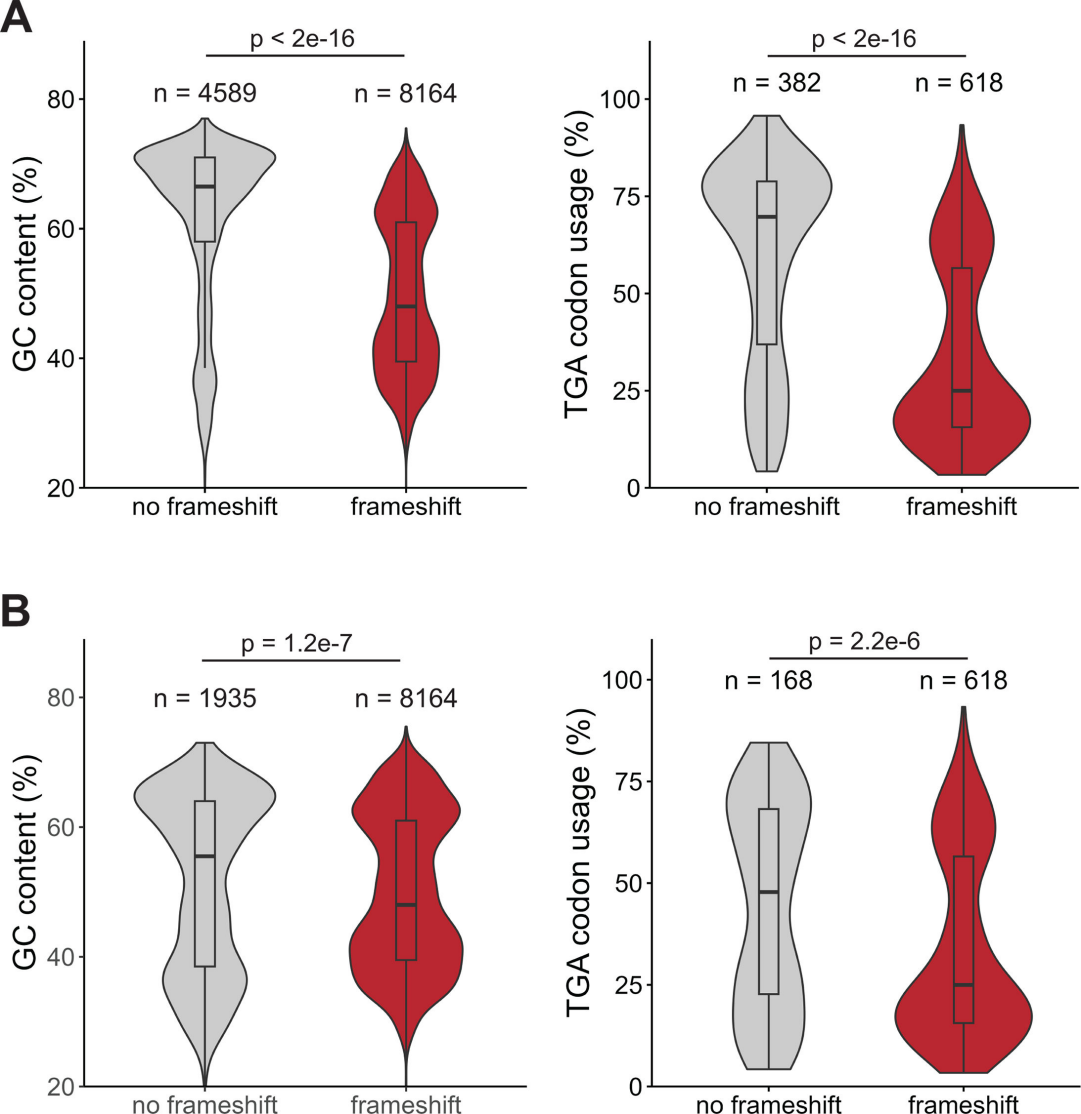
Genomes that lack the programmed frameshift motif within *prfB* have significantly higher GC content and TGA stop codon usage. (**A**) GC content and TGA stop codon usage of a subset of 1,000 random genomes separated by genomes with and without the *prfB* frameshift motif. (**B**) Distributions of GC content and TGA stop codon usage of the random subset without Actinomycetota genomes. *P*-values indicate the results of a Welch two-sample *t*-test.

We next explored genome characteristics of organisms that do not utilize the programmed frameshift in *prfB*. We found that genomes that lack the programmed frameshift motif have significantly higher GC content than genomes with the motif (62% average GC, *P* < 2.2e−16) (Fig. 6A). Our finding remains significant even with the removal of the well-represented high-GC Actinomycetota genomes (*P* < 1.1e−07) (Fig. 6B). GC content positively correlates with RF2-specific TGA codon usage (33) (Fig. S3). Therefore, we hypothesized that organisms lacking RF2 autoregulation would also encode more RF2-specific stop codons. To investigate this, we compared terminal stop codon usage between genomes with and without the programmed frameshift for a random subset of 1,000 genomes. Genomes that lost the *prfB* frameshift motif have significantly higher RF2-specific TGA terminal stop codon usage than genomes that retained the motif (*P* < 2.2e−16) (Fig. 6A). Again, our findings are significant even when Actinomycetota genomes are excluded (*P* < 2.2 e−06) (Fig. 6B). Therefore, a higher demand for RF2 due to increased RF2-specific TGA stop codon usage may help explain the loss of the RF2 autoregulation in some species.

### Ribosomal frameshifting is inefficient at the *prfB* programmed frameshift motif in the actinomycete *Mycobacterium smegmatis*

**Fig 7:**
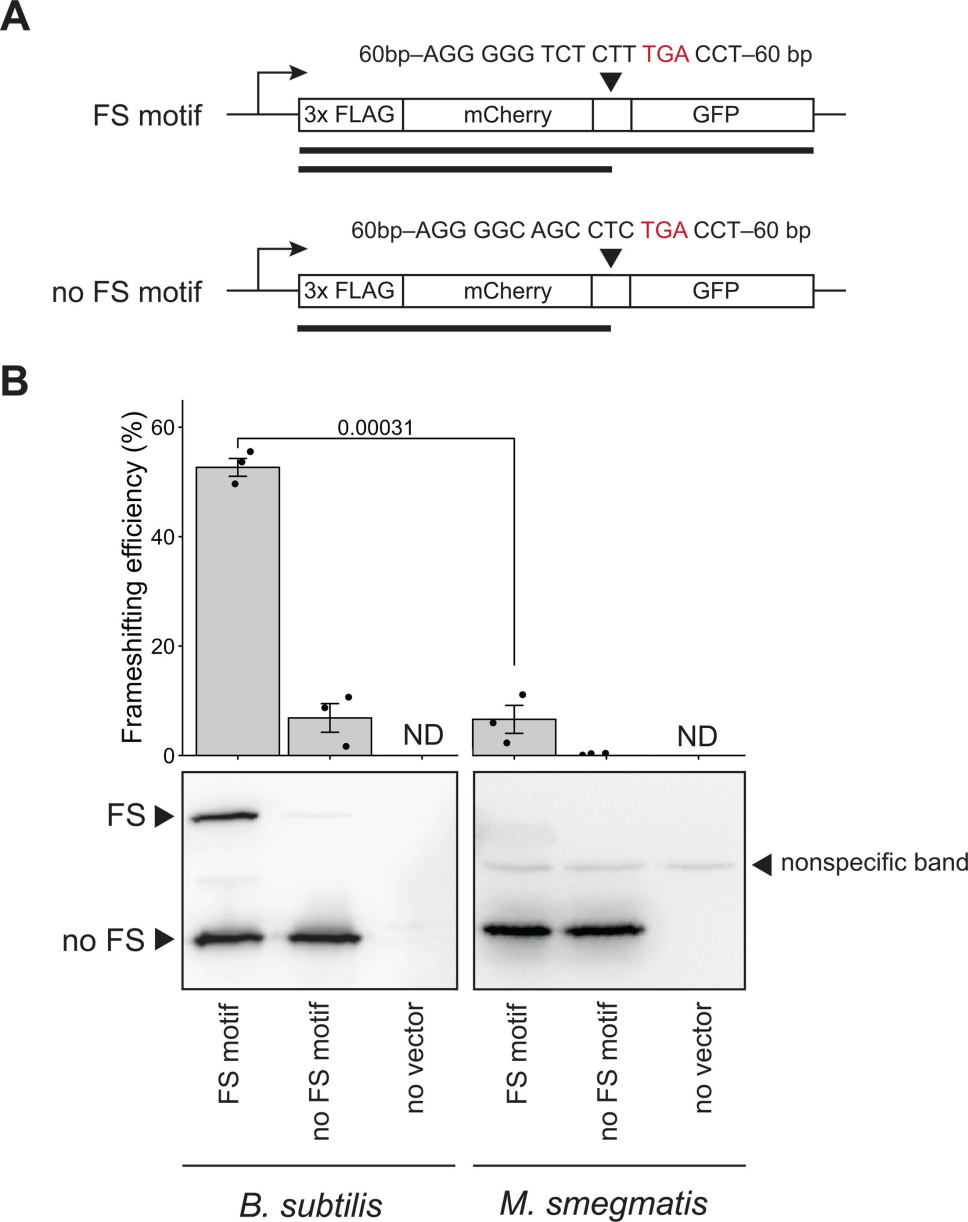
*Mycobacterium smegmatis* cannot efficiently frameshift at the canonical *prfB* frameshift motif. (**A**) Schematics for analogous *prfB* frameshifting reporters in *B. subtilis* and *M. smegmatis*. The “FS motif” construct contains the canonical *prfB* frameshift motif from *B. subtilis* including the premature, in-frame TGA stop codon (highlighted in red) and 60 bp upstream and downstream of the motif. The “no FS motif” construct contains only the premature, in-frame TGA stop codon. The constructs for *M. smegmatis* are codon optimized, except for the *prfB* frameshift motif shown in the figure to ensure efficient translation of the rest of the protein. A larger protein product is produced if frameshifting occurs at the motif, and a smaller protein product is produced if frameshifting does not occur at the motif. (**B**) Western blots representative of three biological replicates showing both frameshifted and termination products resulting from the expression of the described constructs in *B. subtilis* and *M. smegmatis*. Wild-type “no vector” control strains without a frameshifting reporter are included for each species and are shown in lane 3 to show background and non-specific bands. Frameshifting efficiency was calculated based on relative band intensities and is shown above the blot. Frameshifting efficiency = frameshifted protein band intensity/(frameshifted + non-frameshifted protein band intensity). “No vector” controls do not contain reporters and did not produce detectable bands (ND, not detectable). *P*-values indicate the results of a Welch two-sample *t*-test.

No surveyed Actinomycetota genomes (*n* = 2,658) contain the *prfB* frameshift motif. Therefore, we hypothesized that Actinomycetota may exhibit poor frameshift efficiency at the motif. To assay frameshifting efficiency, we compared the frameshifting efficiencies of an organism that natively contains the frameshift motif, *B. subtilis*, and an actinomycete that lacks the motif, *Mycobacterium smegmatis*. We designed analogous constructs for *B. subtilis* and *M. smegmatis* that contain two fused protein sequences (encoding mCherry and GFP) separated by the *prfB* frameshift motif from *B. subtilis* (denoted as “FS motif”). We included ~60 bp upstream and downstream of the frameshift motif to ensure that the sequence context surrounding the frameshift motif is also maintained. In *M. smegmatis*, the fluorescent proteins and the 60 bp regions upstream and downstream of the *prfB* motif were codon optimized for *M. smegmatis* to avoid ribosomes stalling at rare codons. As a control for production of the truncated protein, we used a construct identical to the *prfB* FS motif construct, including the premature TGA stop codon but lacking the frameshift motif elements (denoted as “no FS motif”). Construct schematics are depicted in Fig. 7A.

Upon expressing the described constructs *in vivo*, we observed that ribosomal frameshifting occurs at the *prfB* motif with an efficiency of 52.7% ± 2.8% in *B. subtilis* (Fig. 7B). In contrast, *M. smegmatis* exhibits ribosomal frameshifting at the motif with an efficiency of 6.6% ± 4.4% (Fig. 7B). As expected, neither organism exhibits strong ribosomal frameshifting at the *prfB* variant that lacks the frameshift motif elements (Fig. 7B). The abnormally low frameshift efficiency in *M. smegmatis* at the *prfB* motif supports the idea that members of Actinomycetota have lost this form of RF2 autoregulation because they are unable to efficiently utilize the programmed frameshift motif.

## DISCUSSION

Autoregulation of RF2 levels by a frameshift in *prfB* is a textbook example of programmed ribosomal frameshifting, and the molecular mechanism underlying the programmed frameshift has been precisely characterized in *E. coli* (12, 14). Using >12,000 bacterial genomes and referencing a recently updated bacterial phylogenetic tree (25), we present evidence that this regulatory frameshift motif is highly conserved and was likely present in the ancestral *prfB*. We find that loss of the programmed frameshift highly correlates with RF2-specific stop codon usage. This finding is consistent with the expectation that the requirement for higher RF2 levels will select for inactivation of an autoregulatory mechanism that reduces RF2 expression.

Our analysis of the programmed frameshift elements in *prfB* is consistent with previous reports analyzing fewer than 300 genomes (20–22) and is in perfect agreement with the highly characterized mechanistic features of the ribosomal frameshift. In particular, the SD-like sequence, the spacing between the SD-like sequence and the premature stop codon, the slippery sequence preceding the stop codon, and the C nucleotide immediately following the stop codon are almost universally conserved. Most striking, the identity of the premature stop codon is TGA in >99% of surveyed genomes and is TAA in all remaining genomes surveyed. There is evidence that TAA may be primarily recognized by RF2 in most bacterial taxa (37–41), with the exception of *E. coli* K-12 strains, which encode a less efficient RF2 (39). Therefore, even *prfB* genes containing a premature TAA stop codon would likely be subject to regulation by RF2 levels. In further support of the necessity for the conservation of each frameshifting element, including the stop codon identity, previous work shows that altering any of the elements significantly decreases the frameshifting efficiency in *E. coli* (14). The precise conservation of each of the frameshifting elements throughout the bacterial domain suggests that nearly all bacteria that autoregulate RF2 levels do so by the same molecular mechanism.

Previous work postulated that the ancestral *prfB* lacked the programmed ribosomal frameshift motif. This hypothesis was informed by an understanding of the bacterial tree as being rooted closest to Thermotogota and the absence of the motif in *prfB* sequences from this phylum. We similarly did not detect the motif in *prfB* sequences in Thermotogota (Fig. 3A). However, the overwhelming majority of *prfB* sequences of all phyla in proximity to Thermotogota do indeed utilize the programmed frameshift (Fig. 3A). Since current models place the root of the bacterial tree in the neighborhood of Fusobacteria, and the majority of taxa in this neighborhood, except Thermotogota, use the programmed frameshift in *prfB*, it is likely and parsimonious that the ancestral *prfB* was regulated by a programmed ribosomal frameshift.

The conservation of the programmed ribosomal frameshift motif in *prfB* suggests that it imparts a strong selective advantage. Consistent with this prediction, we found that overexpressing RF2 from *prfB* without the programmed frameshift motif was toxic to *B. subtilis* at 30°C whereas overexpressing RF2 from *prfB* encoding the autoregulatory motif caused no noticeable growth defect compared to wild-type cells (Fig. 4B). In contrast, when we overexpressed RF1 (*prfA*) from an analogous plasmid, we observed a modest but less severe growth defect than the defect caused by RF2 overexpression without autoregulation (Fig. 4B). In support of our findings, we identified only 34 genomes in our data set with *prfA* pseudogenes that would require a frameshift to make the full-length RF1 protein. Even in these genomes, there was no compelling evidence that the *prfA* sequences would utilize a programmed frameshift. Our data suggest that RF2 is more dangerous to the cell than RF1, which necessitates tight autoregulation for RF2 but not RF1.

Having determined the importance of *prfB* autoregulation and that the ancestral *prfB* likely encoded the programmed frameshift motif, we next examined the genome characteristics of taxa that lost the frameshift motif. We found that bacteria that have lost the programmed frameshift motif in *prfB* had significantly higher RF2-specific stop codon usage (*P* < 2.2e^−16^) (Fig. 6A). Even when we excluded Actinomycetota, which make up a large proportion of genomes that do not encode the motif, this finding was still highly significant (*P* = 2.2e^−06^) (Fig. 6B). Bacterial release factor concentrations correlate with cognate stop codon usage (33, 42). The direction of causality is unknown, but a recent *in silico* study proposed that release factor concentrations adapted to stop codon usage (43). High GC content strongly correlates with high RF2-specific TGA stop codon usage but not with TAG stop codon usage (Fig. S3) (33). Therefore, a likely explanation for RF2 autoregulation loss is that high GC content and high RF2-specific stop codon usage increased the demand for RF2, and RF2 autoregulation was subsequently lost to satisfy this demand.

Across the bacterial tree of life, the most notable motif loss event occurred in the Actinomycetota ancestor. None of the 2,658 Actinomycetota genomes in our survey encoded the programmed frameshift motif in *prfB*. Therefore, we hypothesized that Actinomycetota may exhibit poor ribosomal frameshifting efficiency at this motif. In support of this hypothesis, when we provided *M. smegmatis* with a reporter encoding the *prfB* frameshift motif, the frameshift efficiency was ~6% (Fig. 7B). Several factors are known to affect frameshifting at this motif *in vivo* in *E. coli* and *in vitro*, including relative RF2 levels and near-cognate tRNA concentrations (44–46), as well as structural aspects like tRNA positioning (18) and binding of the anti-Shine-Dalgarno sequence of the 16S rRNA to the Shine-Dalgarno-like sequence within *prfB* (14, 19). We cannot conclude from our results whether *M. smegmatis* ribosomes are less able to frameshift at the *prfB* frameshift motif due to ribosome structure differences (47–49) or high cellular RF2 concentrations, which would favor termination at the TGA stop codon over frameshifting. In either case, these results suggest that encoding the ribosomal frameshift motif in *prfB* would be detrimental in *M. smegmatis* because it may result in decreased RF2 levels. Interestingly, the only other organism reported to have similarly low frameshifting efficiency at the *prfB* motif is *Flavobacterium johnsoniae,* which rarely uses Shine-Dalgarno sequences during translation initiation (50, 51). Surprisingly, *F. johnsoniae* retains the *prfB* frameshift motif despite its low frameshifting efficiency. The low efficiency may be tolerated because the *F. johnsoniae* genome has an unusually low proportion of RF2-specific stop codons (7% of all stop codons) (50), and therefore a low demand for RF2.

Although RF2-specific stop codon usage is highly correlated with loss of the motif, there are notable exceptions. In particular, Mycoplasmatota have low genomic GC content and low TGA stop codon usage, but Mycoplasmatota genomes that encode *prfB* all lack the frameshift motif (Fig. 5). The ancestral Mycoplasmatota underwent extreme genome reduction, and therefore, many species in the phylum have lost *prfB,* instead decoding UGA as tryptophan (32, 52). It is possible that in lineages that retain *prfB*, RF2 may be actively decaying and therefore lowly expressed, even without autoregulation. Therefore, RF2 autoregulation is not required in these lineages.

Our work supports a model in which the programmed ribosomal frameshift motif in *prfB* was present in the last common ancestor of bacteria and autoregulates RF2 expression in most bacterial species. In many species that have lost the motif, it is likely that high TGA stop codon usage increased demand for RF2, and so RF2 autoregulation is no longer required. In contrast, increased expression of RF2 may be deleterious in organisms with lower TGA stop codon usage, as we observed in *B. subtilis*. It is notable that RF2 is more toxic than overexpression of RF1 (Fig. 4) and therefore may require special regulation. Indeed, the stress response to RF2 versus RF1 overexpression has been documented (53). While our work offers a comprehensive survey of the evolution of RF2 autoregulation, future studies are needed to determine why uncontrolled RF2 expression is uniquely toxic in some bacteria.

## MATERIALS AND METHODS

### Strains and media

All strains were derived from *B. subtilis* 168 *trpC2* and *M. smegmatis* MC^2^ 155. *B. subtilis* was grown shaking in lysogeny broth media at 37°C, and *M. smegmatis* was grown shaking in 7H9 Middlebrook media at 37°C as indicated. Antibiotics were used at final concentrations of 5 µg/mL chloramphenicol, 20 µg/mL kanamycin, and 1× MLS (250 µg/mL lincomycin and 10 µg/mL erythromycin). Plasmids used in this study are described in Table 1, and novel plasmid sequences are available on the project GitHub at https://github.com/cassprince/prfB_evolution/blob/main/data/plasmid_sequences.fasta. All experiments were performed in biological triplicate.

**TABLE 1:** Strains, plasmids, and primers

Strain, plasmid, or primer	Description or sequence	Source or reference
Strains		
HAF1	*B. subtilis* wild type 168 trpC2	(54)
HAF477	*M. smegmatis* wild type MC2 155	Kenneth Keiler
CP203	168 trpC2 ECE743 empty vector	This study
CP73	168 trpC2 ECE743 *P_xylA_-1*×*FLAG-B. subtilis prfB*	This study
CP215	168 trpC2 ECE743 *P_xylA_-1*×*FLAG-B. subtilis prfB* with recoded frameshift motif	This study
CP266	168 trpC2 ECE743 *P_xylA_-1*×*FLAG-B. subtilis prfA*	This study
CP109	168 trpC2 pHF328 *sacA*::*P_hyperspank_*-3×*FLAG-mcherry-gfp*	This study
CP127	168 trpC2 pHF328 *sacA*::*P_hyperspank_*-3×*FLAG-mcherry-gfp-B. subtilis prfB* frameshift motif-*gfp*	This study
CP271	168 trpC2 pHF328 *sacA*::*P_hyperspank_*-3×*FLAG-mcherry-gfp-B. subtilis prfB* recoded frameshift motif-TGA-*gfp*	This study
CP252	MC2 155 pMV306hsp *P_hsp60_-3*×*FLAG-mcherry-gfp* codon optimized for *M. smegmatis*	This study
CP253	MC2 155 pMV306hsp *P_hsp60_-3*×*FLAG-mcherry-B. subtilis prfB* frameshift motif-*gfp* codon optimized for *M. smegmatis*	This study
CP267	MC2 155 pMV306hsp *P_hsp60_-3*×*FLAG-mcherry-B. subtilis prfB* recoded frameshift motif-TGA-*gfp* codon optimized for *M. smegmatis*	This study
Plasmids		
ECE743	Empty vector, ori1030, XylR-P_xylA_ upstream of MCS, amp^R^, mls^R^ (replicative)	(55)
pHF328	pDR111 P_hyperspank_ and MCS cloned into ECE174 backbone at the BamHI and EcoRI sites. Integration at *sacA*	This study
pCP66	ECE743 *P_xylA_-1*×*FLAG-B. subtilis prfB* (replicative)	This study
pCP209	ECE743 *P_xylA_-1*×*FLAG-B. subtilis prfB* with recoded frameshift motif (replicative)	This study
pCP254	ECE743 *P_xylA_-1*×*FLAG-B. subtilis prfA* (replicative)	This study
pCP105	pHF328 *sacA*::*P_hyperspank_-3*×*FLAG-mcherry-gfp*	This study
pCP125	pHF328 *sacA*::*P_hyperspank_-3*×*FLAG-mcherry-B. subtilis prfB* FS*-gfp*	This study
pCP269	pHF328 *sacA*::*P_hyperspank_-3*×*FLAG-mcherry-B. subtilis prfB* no FS motif-*gfp*	This study
pCP234	pMV306hsp *P_hsp60_-3*×*FLAG-mcherry-gfp* codon optimized for *M. smegmatis* (replicative)	This study
pCP238	pMV306hsp *P_hsp60_-3*×*FLAG-mcherry-B. subtilis prfB* FS-*gfp* codon optimized for *M. smegmatis* (replicative)	This study
pCP258	pMV306hsp *P_hsp60_-3*×*FLAG-mcherry-B. subtilis prfB* no FS-*gfp* codon optimized for *M. smegmatis* (replicative)	This study
Primers		
CP70-F	5′-GGTGATGTACTTACTATATGAAATAAAATGCATCTGTAGAATTC-3′	This study
CP71-R	5′-GGGCCTCCTTTGATTCGAGGTCAAAGAGA-3′	This study
CP72-F	5′-TCTCTTTGACCTCGAATCAAAGGAGGCCC-3′	This study
CP73-R	5′-CATGATTACGCCAAGCTTGCATGCTTATGAAAGC-3′	This study
CP76-F	5′-ATAACAATTAAGCTTGGAGGAAAAAAAATGGATTATAAAGACGACGACG-3′	This study
CP77-F	5′-ACCTTTAGACAGACCTGAATTCGAGCTCGGTACCC-3′	This study
CP78-F	5′-CCGAGCTCGAATTCAGGTCTGTCTAAAGGTGAAGAACTG-3′	This study
CP79-R	5′-TTGCATGCGGCTAGCTTATTTGTAGAGCTCATCCATGCCG-3′	This study
CP120-F	5′-ATGATGATGATAAAGTCGACGTGTTAGACCGTTTAAAATCAATTGAAGAACG-3′	This study
CP121-R	5′-ACCATGATTACGCCAAGCTTTTAACCTTCCGACTGCTGAAGCTTGC-3′	This study

### Release factor overexpression

Overnight cultures were normalized to an OD600 of 0.05. For spot plates, 10-fold serial dilutions were spotted onto LB containing 1× MLS and 0%, 1%, or 5% xylose and incubated at 30°C for 24 hours. For measurement of overexpression levels, cultures were grown in LB containing 1× MLS at 30°C to an OD of 1 and subsequently induced with 0%, 1%, or 5% xylose. Cells were harvested and pelleted after 2.5 hours of induction.

### Frameshift reporter lysates and western blots

*M. smegmatis* overnight cultures were normalized to an OD600 of 0.2. Cells were then harvested and pelleted after 12 hours. *B. subtilis* overnight cultures were normalized to an OD600 of 0.05. Reporter expression was induced with 1 mM IPTG when cultures reached an OD600 of 1. Cells were then harvested and pelleted after 30 minutes of induction. *B. subtilis* and *M. smegmatis* cell pellets were treated with lysis buffer (10 mM Tris, pH 8, 50 mM EDTA, 1 mg/mL lysozyme) for 10 minutes at 37°C. *M. smegmatis* cells were further lysed using bead beating for five cycles of 20 seconds at 4,350 rpm with 3 minutes on ice between cycles. All lysates were mixed with SDS loading dye, heated at 90°C for 5 minutes, and cooled on ice. For *M. smegmatis*, the protein levels of the full-length reporter were approximately 15× greater than those of the experimental *prfB* frameshifting reporters based on band intensity. Therefore, the lysates for the full-length reporter strain were diluted 15× to normalize the protein levels between reporters. Proteins were then resolved on a 12% SDS-PAGE gel for 70 minutes at 150 V. To measure *prfB* overexpression levels and reporter frameshifting levels, proteins were transferred from SDS-PAGE gels to a PVDF membrane (BioRad) for 100 minutes at 300 mA. The membrane was blocked in 3% bovine serum albumin (BSA) overnight at 4°C. Anti-FLAG antibody conjugated to horseradish peroxidase (Sigma SAB4200119) was added to the BSA for 1.5 hours at room temperature. The membrane was washed with PBS-T three times for 5 minutes at room temperature and developed with ECL substrate and enhancer (Biorad 170-5060). Band intensities were quantified using ImageJ v1.53k (56). *P*-values for differences in band intensity were calculated with the R stats v4.2.2 package using a Welch two-sample *t*-test.

### Release factor gene sequence acquisition and analyses

*prfB* and *prfA* nucleotide sequences were downloaded from all representative prokaryotic genomes in the NCBI RefSeq database as annotated by the NCBI Prokaryotic Genome Annotation Pipeline (23). Only genomes with CheckM (57) contamination scores below 10% were used, resulting in 12,753 genomes. The NCBI accession numbers, species names, and taxonomy for all genomes used can be found in Table S1. Sequences were aligned using MAFFT v7.453 (58). Python scripts were used to identify premature stop codons by searching for any stop codon that was in-frame but not found in the final three nucleotides of the sequence. The surrounding region was then extracted, and the identity of the stop codon was recorded. If no premature stop codon was found, the expected region of the frameshift motif (based on multiple sequence alignment) was extracted. The scripts utilized the Biopython v1.78 (59) package for sequence manipulation. The extracted regions were converted to sequence logos using the logomaker v0.8 package (60).

### Phylogenetic analyses

16S rRNA sequences were identified and acquired using BLAST v2.13.0. Sequences were aligned using MAFFT v7.453 (58). The alignments were applied to FastTree v2.1.11 (61) to infer a maximum likelihood tree. Trees were visualized using the ggtree v3.6.2 package (62). FastTree produces unrooted phylogenies, so trees were midpoint rooted using the phangorn v2.11.1 package (63). The simplified tree in Fig. 3A was produced by randomly selecting a representative genome for each phylum and subsetting the large 16S tree in Fig. 3B. Random genomes were selected from the entire data set by grouping the data by phylum and using the “sample_n” function from the dplyr v1.1.4 package. The identities of the randomly selected genomes can be found in Table S3. Taxonomic classification was assigned to genomes using the NCBI Taxonomy database (64) and taxonkit v0.14.1 (65). GC content for each genome was downloaded from NCBI. To determine terminal stop codon usage, all coding sequences were downloaded as annotated by NCBI PGAP for a random subset of 1,000 genomes. The list of genomes in the subset can be found in Table S2. A novel Python script recorded the last three nucleotides of each coding sequence per genome, utilizing the biopython v1.78 package for sequence manipulation. *P*-values for differences in GC content and terminal stop codon usage between “frameshift” and “no frameshift” genomes were calculated with the R stats v4.2.2 package using a Welch two-sample *t*-test.

## Data Availability

Data for all genomes surveyed can be found in Tables S1 and S2. Scripts for data acquisition and analyses are available on GitHub at https://github.com/cassprince/prfB_evolution.

## SUPPLEMENTAL MATERIAL

Fig. S1 mbio.01055-25-s0001.eps TAA is poorly represented and randomly distributed as the premature stop codon in the frameshift motif.

Fig. S2 mbio.01055-25-s0002.eps Percent of genomes containing the *prfB* frameshift motif across phyla.

Fig. S3 mbio.01055-25-s0003.eps TGA stop codon usage increases with GC content.

Legends mbio.01055-25-s0004.docx Legends for Figures S1 to S3.

Tables S1 to S3 mbio.01055-25-s0005.xlsx Genome metadata, stop codon usage, and accession numbers.

Captions mbio.01055-25-s0006.docx Captions for Tables S1 to S3.

ASM does not own the copyrights to Supplemental Material that may be linked to, or accessed through, an article. The authors have granted ASM a non-exclusive, world-wide license to publish the Supplemental Material files. Please contact the corresponding author directly for reuse.
