# Supplementary material for: Conservation and evolution of the programmed ribosomal frameshift in prfB across the bacterial domain
Source: mBio. 2025 Aug 18;16(9):e01055-25. doi: 10.1128/mbio.01055-25 (PMC12421855; doi:10.1128/mbio.01055-25)
Supplement: Legends — for Figures S1 to S3. [file mbio.01055-25-s0004.docx]

**Figure S1. TAA is poorly represented and randomly distributed as the premature stop codon in the frameshifting motif.** 16S maximum-likelihood phylogenetic tree from Figure 3B showing the distribution of premature stop codons in *prfB*. Phyla with more than 10 available and high-quality reference genomes are shown.

**Figure S2. Percent of genomes containing the *prfB* frameshifting motif across phyla.** A subtree of the large 16S tree was created using a random single representative genome for each phylum with more than 10 genomes. The number of analyzed genomes per phylum and values for the percentage of genomes with the frameshift are located to the right of each bar. Darkened circles indicate bootstrap values for each node.

**Figure S3. TGA stop codon usage increases with GC content.** Terminal stop codon usage in all coding sequences for a subset of 1000 random reference genomes plotted verses the genomic GC content. Linear regressions and equations for the line of best fit are shown for each stop codon. TAA stop codon usage strongly correlates with GC content (R^2^ = 0.89) and decreases with increasing GC content, as indicated by the negative slope of the regression line. TGA stop codon usage strongly correlates with GC content (R^2^ = 0.83) and increases with increasing GC content, as indicated by the positive slope. TAG stop codon usage does not correlate with GC content (R^2^ < 0.01). Each genome has three points, one for each of the three stop codons.
