# Supplementary material for: Conservation and evolution of the programmed ribosomal frameshift in prfB across the bacterial domain
Source: mBio. 2025 Aug 18;16(9):e01055-25. doi: 10.1128/mbio.01055-25 (PMC12421855; doi:10.1128/mbio.01055-25)
Supplement: Captions — for Tables S1 to S3. [file mbio.01055-25-s0006.docx]

**Supplemental table legends**

**Table S1.** Metadata of the 12,571 genomes used in this study.

**Table S2.** Total stop codon usage for the random subset of 1,000 genomes in Figure 6.

**Table S3.** Accession numbers for random representatives of phyla used to build the trees in Figure 3A and S2.
